# The endophytic fungi *Metarhizium*, *Pochonia*, and *Trichoderma*, improve salt tolerance in hemp (*Cannabis sativa* L.)

**DOI:** 10.1371/journal.pone.0325559

**Published:** 2025-06-11

**Authors:** Shasha Hu, Michael J. Bidochka

**Affiliations:** 1 Department of Biological Sciences, Brock University, St. Catharines, Ontario, Canada; Universitat Jaume 1, SPAIN

## Abstract

Colonization of plants by fungal endophytes can improve plant growth and can assist in adaptation to biotic and abiotic stresses. The fungal endophytes *Metarhizium robertsii* and *Pochonia chlamydosporia* were previously shown to improve hemp growth. Here, the impact of three fungal endophytes, *M. robertsii*, *P. chlamydosporia* as well as *Trichoderma harzianum* on hemp was investigated under treatment with 300 mM NaCl as a salinity stress and reduced watering volume as a drought stress. Plant growth parameters, a lipid oxidation indicator, leaf porphyrins together with the abiotic stress responses genes were assessed in hemp with or without fungal colonization under normal and stressed conditions. Under salinity stress, the growth of hemp was ameliorated by the application of *Metarhizium*, *Pochonia*, or *Trichoderma* in the soil. The increased production of malondialdehyde (MDA) and the reduction in porphyrins in hemp under salinity stress were restored in the presence of fungal endophytes. Under drought stress, the aboveground growth of hemp was recovered by the application of *Metarhizium* together with the reduced production of porphyrins. The stress related gene *CsNAC3* showed decreased expression during fungal application compared with uninoculated hemp under salinity or drought treatment. Colonization of *Metarhizium*, *Pochonia* or *Trichoderma* improved salt stress tolerance in hemp and this was accompanied by a reduction in oxidative stress.

## Introduction

Abiotic stresses can reduce plant growth and development. Responses to drought and salinity have been observed in many plants [1] and includes induced reactive oxygen species (ROS) formation that damages cell structure/function [2], lowered photosynthetic efficiency [3], and expression of stress response genes [4]. Symbiosis with beneficial fungal endophytes may mitigate abiotic stress in plants and the improvement of plant growth during abiotic stress treatment was observed with mycorrhizal fungi [5], rhizobia [6], and plant growth-promoting rhizobacteria [7].

Fungal endophytes are ubiquitous in plants in nature, as well as in agricultural crops. Association of fungal endophytes with host plant roots may benefit plant fitness by promoting plant growth through increased nutrient availability and the enhancement of plant tolerance to biotic stresses, such as pathogens and herbivorous pests [8]. *Metarhizium* spp. are known as insect pathogens but can also form endophytic associations with plants, acting as a biofertilizer [9]. It can colonize the roots of plants and transfer insect-derived nitrogen from infected insects to the plant host in exchange for photosynthate [10,11]. *Pochonia chlamydosporia*, a nematophagous fungus, is monophyletically related to *Metarhizium* and can also colonize plant roots and improve plant growth [12]. *Trichoderma harzianum* is a beneficial biocontrol agent utilized in agriculture for its ability to inhibit pathogenic fungi and promote plant growth [13].

Hemp is a very important industrial crop for the production of fiber, seeds and bioactive cannabinoids [14,15]. Hemp has a naturally high tolerance to soil salinity, and plant transcription factors involved in hemp response to salt stress are well-known [16]. The application of entomopathogenic fungi *Metarhizium* and *Beauveria* was reported to control cannabis aphid (*Phorodon cannabis)* on *Cannabis sativa* [17]. In a previous study, we observed that root colonization by *Metarhizium* and *Pochonia* improved hemp growth [18]. The aim of this study was to examine the influence of the application of fungal endophytes *M. robertsii*, *P. chlamydosporia*, and *T. harzianum* on the resistance of hemp to salinity and drought stress. Our hypothesis was that *Metarhizium*, *Pochonia* and *Trichoderm*a were able to ameliorate the negative effects of salinity stress on the growth of hemp in comparison to non-endophytic controls under the same conditions. We observed that the alleviation of salinity stress in hemp during endophytic association was accompanied by a reduction in oxidative stress markers in the plant.

## Materials and methods

### Fungal isolates

The fungal isolates used in this study were *Metarhizium robertsii* ARSEF 2575, *Pochonia chlamydosporia* and *Trichoderma harzianum* UAMH 4162. *P. chlamydosporia* was isolated from an ant hill in Ontario Canada [19]. *T. harzianum* UAMH 4162 was isolated in Alberta Canada and was obtained from the University of Alberta Microfungus Herbarium (UAMH) center. The fungal isolates were cultured on Potato Dextrose Agar (PDA), 24 g L^-1^ Potato Dextrose Broth (Bioshop Canada Inc.) and 15 g L^-1^ agar (Fisher Chemical), at 27 °C for 14 days in dark. Fungal conidia were obtained by flooding a PDA culture with a 0.01% Triton X-100 solution. The suspension was passed through a funnel containing glass wool to obtain a conidial suspension (1.0 × 10^7^ conidia mL^-1^). A hemocytometer was utilized to count and adjust conidial concentrations.

### Plant growth conditions and treatment of salinity stress and drought stress.

Hemp seeds (*Cannabis sativa* cv. ‘Anka’) (UniSeeds Inc.) were washed with 10% bleach for 10 min and subsequently rinsed with sterile distilled water to remove the bleach. The surface-sterilized hemp seeds (2 g) and 25 mL sterilized distilled water were placed in a 50 mL Falcon tube. The immersed hemp seeds were shaken at room temperature with the speed of 3 rpm on a rocking platform shaker (VWR International). Potential seed contamination was assessed by placing an aliquot of the water on PDA plates. After 2 days of shaking in water, the hemp seeds were germinated in autoclaved soil (ASB Grower Mix 15% perlite, JVK.) for 6 days at room temperature. The composition of the soil was 85% Canadian sphagnum peat moss with 15% coarse perlite. The ratio of nutrient elements, nitrogen, phosphorus, and potassium in this soil is 0.15: 0.10: 0.20. The soil pH ranged from 5.0 to 5.7. Before application, the moist soil was autoclaved at 121 °C for 20 min. The soil was autoclaved three times with intervals of more than 24 hours. The germinated seedlings were planted into autoclaved moist soil (JVK) in a black Azalea round pot (TERIS Corporation; 15.24 cm in diameter by 10.8 cm in height). The pots were sterilized under UV light for 3 h prior to use.

Each germinated hemp seedling with visible roots was planted in an individual pot. Then the planted hemp seedling was subsequently drenched with 5 mL (1.0 × 10^7^ conidia mL^-1^) of a fungal conidial suspension only once at the seedling stage. The un-inoculated control hemp was drenched with the same amount of 0.01% Triton X-100. The hemp seedlings were grown in a greenhouse with growth conditions of 28°C daytime and 18°C nighttime with a photoperiod of 18 hours a day. The relative humidity was maintained between 60% and 80%. The control hemp without stress treatment was watered with 100 mL modified 50% MMN solution every two days starting from 3^rd^ day after planting in the pot until the end of experiment. The modified 50% MMN solution contained 222 mL 50% MMN solution (0.05 g CaCl_2_, 0.025 g NaCl, 0.05 g KH_2_PO_4_, 0.5 g (NH_4_)_2_HPO_4_, 0.15 g MgSO_4_ 7H_2_O, 1 mg FeCl_3_ 6H_2_O, 5 g glucose monohydrate and 10 mL trace elements solution (all weights per 1 L)), 3.33 g 95% NH_4_NO_3_ and 778 mL H_2_O. Trace elements solution contained 3.728 g KCl, 1.546 g H_3_BO_3_, 0.845g MnSO_4_ H_2_O, 0.05 g ZnSO_4_ 7H_2_O, 0.0125 g CuSO_4_, 0.05 g (NH_4_)_6_Mo_7_O_24_ 4H_2_O (all weights are per 1 L) [11]. The hemp plants under salinity stress treatment were watered with 100 mL modified 50% MMN solution containing an additional 1.75 g NaCl (approx. 300 mM NaCl) every two days from day 15 to day 35. The salinity stress of 300 mM NaCl was chosen based on a pre-experimental result (unpublished) and previous research [20], which showed reduced, but not detrimental, growth of hemp at this concentration. For hemp plants under drought stress, the volume of the modified 50% MMN solution was reduced to 10 mL every two days from the seventh watering to the seventeenth watering.

### Measurement of growth parameters

Hemp was harvested and carefully removed from pots on day 36 after inoculation. The hemp leaves were collected and weighed. The hemp plant was rinsed with distilled water and excess water removed on a filter paper before measuring the fresh weight of stem and the roots. Since the hemp leaf arrangement followed opposite phyllotaxy, one half of the leaves from each node were immediately ground to a powder in liquid nitrogen for the analysis of MDA, porphyrins, gene expression, and leaf pigment. The other half was weighed for fresh weight (FW) and then dried for leaf water content measurement.

### Quantification of malondialdehyde (MDA), leaf pigments, and porphyrins

The end product of lipid peroxidation, MDA, was estimated by the colorimetric method using thiobarbituric acid (TBA) [21,22]. Briefly, the powdered hemp leaves were placed into 1% trichloroacetic acid. The homogenate was then centrifuged at 10,000 rpm for 5 min. The supernatant of the extraction and 0.5% (w/v) TBA were heated at 95 °C for 30 min. Then the sample were cooled in an ice bath for 30 min. The absorbance of the supernatant after 5000 rpm for 5 min was measured at 532 nm and 600 nm in a Genesys™ 10S UV-Vis spectrophotometer (Thermo Fisher Scientific), using the following calculation; MDA = 1000 [(A_532_ − A_600_ nm)/155] μg.

The hemp leaf powder was placed into 80% aqueous acetone. The crude extract was centrifuged at 1500g for 5 min. The supernatant was measured at 663.6, 646.6 and 440.5 nm in a Genesys™ 10S UV-Vis spectrophotometer (Thermo Fisher Scientific, USA), using the following calculations; Chlorophyll a = 12.25 A_663.6_–2.55 A_646.6_ (μg/mL), Chlorophyll b = 20.31 A_646.6_–4.91 A_663.6_ (μg/mL), Chlorophyll a + b = 17.76 A_646.6 _+ 7.34 A_663.6_ (μg/mL) and Carotenoid = 4.69 A_440.5_–0.267 Chl a + b (μg/mL) [23–25]. Porphyrin content was also determined [26]. The supernatant was mixed with an equal volume of hexane and vortexed. The mixed solvent was centrifuged at 5000 rpm for 3 min. The lower fraction was used to measure the absorbance at 565, 590 and 628 nm. The following equations were utilized to determine the content of protoporphyrin (PPIX), and magnesium protoporphyrins IX (MGPP); PPIX = 196.25A_575_ − 46.6 A_590_ − 58.68 A_628_ (nmole), MGPP = 61.81A_590_ − 23.77A_575_ − 3.55A_628_ (nmole)

### Soil electrical conductivity measurement

The rhizoplane soil was collected and dried in the oven at 65 °C for more than 72 hours. The soil dry weight was determined when there were no changes of weight for consecutive two weighing. Then 11 mL distilled water was added to 1 g of dry soil. The electrical conductivity was measured in the supernatant after centrifuging at 5000 rpm for 10 min. The electrical conductivity was measured with TK303PLUS Water Quality Meter (TEKCOPLUS).

### Water content in plant leaf and soil humidity

Fresh weight (FW) of the leaves or the harvested soil were measured. For dry weight (DW) measurements, the leaf samples were placed in an oven at 65 °C for more than 72 hours. The dry weight was determined when there were no changes in weight after two consecutive weighings. The leaf water content or soil humidity was measured following [(FW – DW)/FW] × 100.

### Quantitative real-time RT-PCR analysis

The expression of the salt stress induced transcription factor, CsNAC3, was assessed [16]. Hemp leaf RNA (n = 3 for each group) was extracted using the QIAzol Lysis Reagent (Qiagen). After DNase-treatment by RNase-Free DNase (Promega), the RNA concentration was determined spectrophotometrically using a Qubit (Invitrogen). Complementary DNA (cDNA) was obtained by reverse transcribing total RNA with a cDNA reverse transcription kit (Applied Biosystems, Thermofisher Scientific) following manufacturer’s instructions. Real-time PCR was conducted using a SensiFAST™ SYBR No-ROX kit (Bioline) in a volume of 10 μL, including 5 μL 2 × SensiFAST SYBR® No-ROX Mix, 2 μL cDNA, 0.5 μL of each forward and reverse primers (10.0 μM). The sequences of primers are listed in Table 1. The non-template control, no reverse transcriptase control and positive control were included in the test. The PCR protocol included a 2 min initial denaturation step at 95°C, followed by 40 cycles of 5 s at 95°C and 30 s at 70°C. Fluorescence measurements were collected at each polymerization step, then held at 72 °C for 2 min. The melting curve (65–95 °C) was taken at 0.5 °C intervals. PCR products were checked using a melt curve analysis after quantification. The relative expression levels of this gene were normalized against the reference gene, *EF1α* [27] using Bio-Rad CFX Manager software.

**Table 1:** PCR primers tested in this paper.

ID	Gene	Forward/Reverse sequence	Target
XM_030633854	*CsNAC3*	ATGGGTGTACCCGAGATGG TGGTAATACCCATGGGTCAAACTTG	*Cannabis sativa* NAC domain-containing protein
JP452083.1	*EF1*α	TGTTTTGCACGGATCAGTTTG AATGCCGACCGCTACAGTTC	*Cannabis* elongation factor 1 alpha

### Statistical analysis

Data analysis was conducted using GraphPad Prism version10.0.3 for macOS, GraphPad Software, Boston, Massachusetts USA. The data was verified for normal distribution by the Shapiro-Wilk test. The normally distributed data, which passed the F-test, were analyzed by unpaired *t*-tests. The normally distributed data, which failed the F-test, statistically significant differences were determined using unpaired t-test with Welch’s correction. For data not conforming to a normal distribution by Shapiro-Wilk test, the non-parametric Mann-Whitney test for pairwise comparisons was used [28]. However, for results with no statistically significant differences, the possibility of a Type II error cannot be ruled out due to small sample sizes.

## Results

### Fungal application and hemp growth under NaCl stress and drought stress.

**Fig 1:**
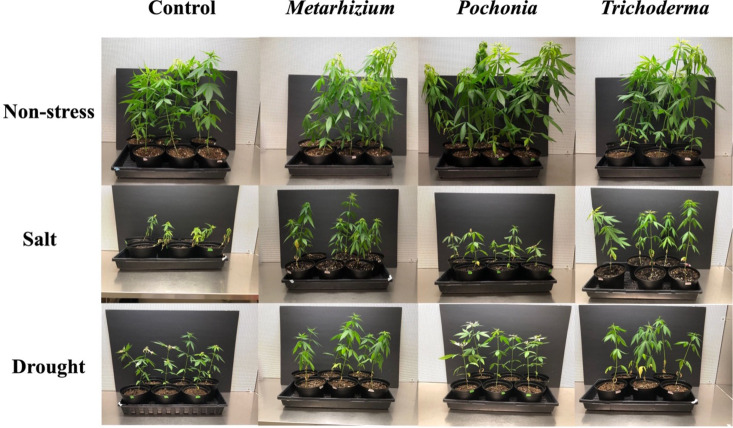
Impact of endophytic fungi on the growth of hemp under non-stress, salt, and drought stress conditions. Control refers to plants grown without endophytic fungi. Plants were grown for 42 days (6 days from seeds to seedlings and 36 days in greenhouse).

**Fig 2:**
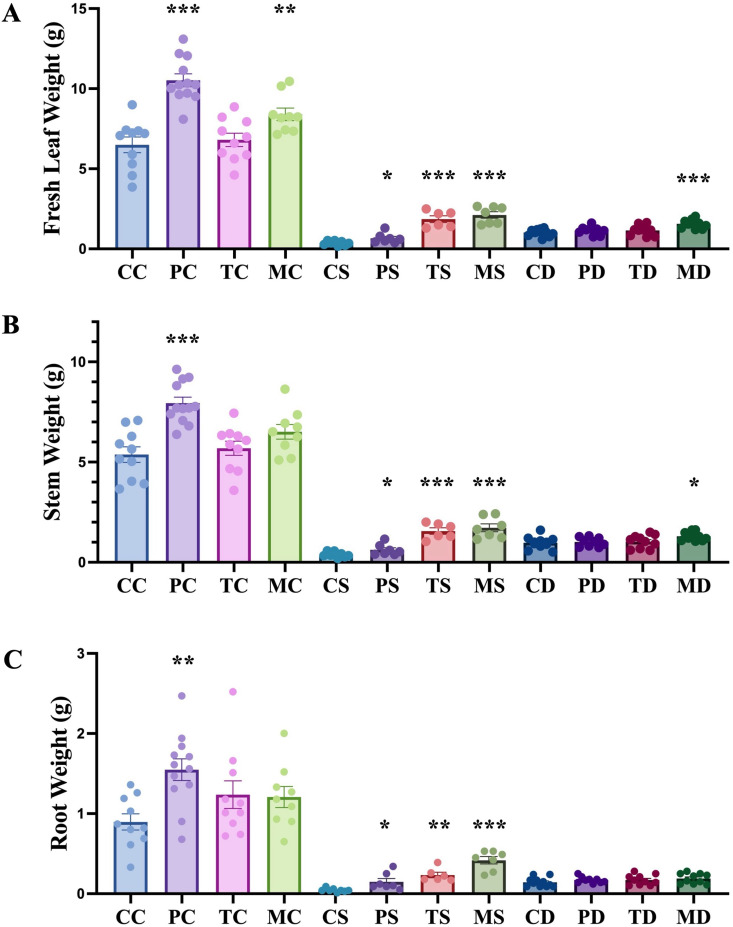
Impact of endophytic fungi on weight of hemp leaf, stem and root, under non-stress, salinity and drought stress conditions. The first letter; C = control, no fungus, P = with *Pochonia chlamydosporia*, T = with *Trichoderma harzianum*, M = with *Metarhizium robertsii*. The second letter; C = control, non-stress, S = salt stress, D = drought stress. Data were analyzed according to the description of statistical analysis. Statistical differences are shown; *P < 0.05, **P < 0.01, ***P < 0.001. Asterisk indicates significant differences in the tested group when compared to that of the un-inoculated control under the same conditions. The dots represent results of the individual biological replicates. Error bars represent standard error of the means.

NaCl stress caused a decrease in fresh leaf weight, stem weight, and root weight of hemp plants (Figs 1 and 2). Application of *P. chlamydosporia*, *T. harzianum*, and *M. robertsii* enhanced hemp growth parameters compared with the uninoculated control plants under salinity stress. Fresh leaf weight decreased from 6.49 ± 0.49 g plant^-1^ (n = 10) in the control to 0.36 ± 0.05 g plant^-1^ (n = 8) (P < 0.0001, unpaired t-test with Welch’s correction) with a 300 mM NaCl treatment without fungal application. Application of *P. chlamydosporia*, *T. harzianum*, and *M. robertsii* with the NaCl stress increased fresh leaf weight to 0.66 ± 0.12 g plant^-1^ (n = 7) (P < 0.05, unpaired t-test with Welch’s correction), 1.85 ± 0.21 g plant^-1^ (n = 6) (P < 0.001, unpaired t-test with Welch’s correction) and 2.12 ± 0.20 g plant^-1^ (n = 7) (P < 0.001, non-parametric Mann-Whitney test), respectively (Fig 2A). The stem weight was reduced by 92.9% (P < 0.0001, unpaired t-test with Welch’s correction) during NaCl treatment in the un-inoculated control. A significant increase in stem weight was observed in hemp treated with *P. chlamydosporia*, *T. harzianum*, and *M. robertsii* with a 63.0% (P < 0.05, unpaired t-test), 309.2% (P < 0.001, unpaired t-test with Welch’s correction) and 351.5% (P < 0.001, unpaired t-test with Welch’s correction) increase when compared to the uninoculated control under salinity stress (Fig 2B). Root weight decreased by 95.2% in uninoculated hemp under salinity stress. The root weight increased by 2.4-fold (P < 0.05, unpaired t-test with Welch’s correction), 4.3-fold (P < 0.01, unpaired t-test with Welch’s correction) and 8.5-fold (P < 0.001, unpaired t-test with Welch’s correction), respectively, with the application of *P. chlamydosporia*, *T. harzianum*, and *M. robertsii* under salinity stress when compared to the uninoculated control with the same treatment (Fig 2C). Increases in hemp growth were observed with the application of all fungal endophytes under salt stress compared with the stressed controls. However, growth parameters of hemp with these fungi under salinity stress were still significantly lower than the un-inoculated controls without stress. The application of *P. chlamydosporia*, *T. harzianum*, and *M. robertsii,* mitigated the adverse effects of NaCl on hemp growth but did not completely recover plant growth (i.e., compared to the non-stress controls).

Decreased growth was also observed in terms of leaf weight (P < 0.0001, unpaired t-test with Welch’s correction), stem weight (P < 0.0001, unpaired t-test with Welch’s correction), and root weight (P < 0.0001, unpaired t-test with Welch’s correction) in uninoculated hemp (n = 11) under drought stress. The application of *M. robertsii* (n = 10) resulted in an increase in the weight of above ground parts, fresh leaf weight by 52.86% (P < 0.0001, unpaired t-test) and stem weight by 33.17% (P < 0.05, unpaired t-test), respectively, compared to the uninoculated control under drought stress (Figs 2A and 2B). Without stressed conditions, the application of *P. chlamydosporia* (n = 12) resulted in an increase in fresh leaf weight (P < 0.0001, unpaired t-test), stem weight (P < 0.0001, unpaired t-test) and root weight (P < 0.01, unpaired t-test) compared to uninoculated control (Fig 2). The application of *M. robertsii* (n = 9) also increased the fresh leaf weight (P < 0.01, unpaired t-test) in hemp under no drought stress conditions (Fig 2A).

The leaf pigments and water content together with soil electrical conductivity and humidity were also measured after harvesting inoculated and un-inoculated hemp plants. An increase in leaf chlorophyll a and carotenoid was observed only in *T. harzianum* colonized hemp when compared to the un-inoculated hemp under salt stress (S1 Fig). The colonization of *P. chlamydosporia* also showed increased production of carotenoid under salt stress (S1 Fig). No differences in leaf water content were observed in hemp with fungi under salt or drought stress compared to the un-inoculated controls (S2 Fig), which could result from the high abiotic tolerance of hemp. The electrical conductivity of plant soil was tested to indicate the soil salt conditions of hemp during the application of fungi under abiotic stress. There were no significant differences of hemp soil after application of fungi in comparison to the uninoculated controls under the salt stress (S3 Fig). These results suggested that the alleviation of salinity stress by fungal application was not related to the changes in the rhizoplane soil salt concentration. The application of fungal endophytes affected soil moisture levels differently under drought stress; an increase in soil moisture was observed with application of *P. chlamydosporia* and a decrease with *T. harzianum* and *M. robertsii* (S4 Fig).

### Regulation of the plant anti-oxidative system

**Fig 3:**
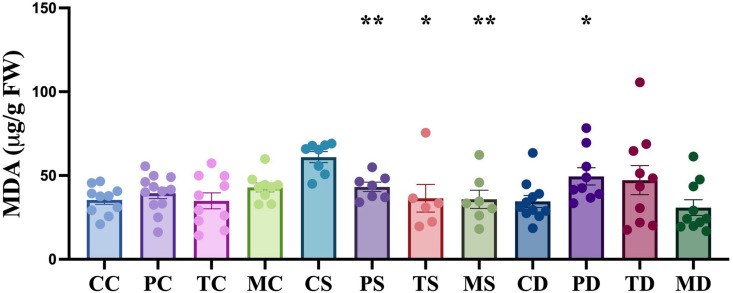
Impact of endophytic fungi on the concentration of malondialdehyde (MDA) in hemp leaves under non-stress, salinity and drought stress conditions. The first letter; C = control, no fungus, P = with *Pochonia chlamydosporia*, T = with *Trichoderma harzianum*, M = with *Metarhizium robertsii*. The second letter; C = control, non-stress, S = salt stress, D = drought stress. Data were analyzed according to the description of statistical analysis. Statistical differences are shown; *P < 0.05, **P < 0.01, ***P < 0.001. Asterisk indicates significant differences in the tested group when compared to that of the un-inoculated control under the same conditions. The dots represent results of the individual biological replicates. Error bars represent standard error of the means.

MDA is a product of lipid oxidation, and no differences in total MDA content was observed in hemp leaves between colonized and non-colonized plants in normal conditions. However, an increase of 72.1% MDA (P < 0.0001, unpaired t-test) was found in uninoculated hemp under salinity stress compared to no stress conditions (Fig 3). Fungal application of *P. chlamydosporia*, *T. harzianum*, and *M. robertsii* significantly decreased MDA content in hemp leaf under salinity stress by 29.1% (P < 0.01, unpaired t-test), 40.3% (P < 0.05, non-parametric Mann-Whitney test) and 41.3% (P < 0.01, unpaired t-test), respectively (Fig 3). There was no significant differences in the amount of MDA in uninoculated control under drought stress compared to no stress conditions.

Influence of fungal application on porphyrins in hemp leaf.

**Fig 4:**
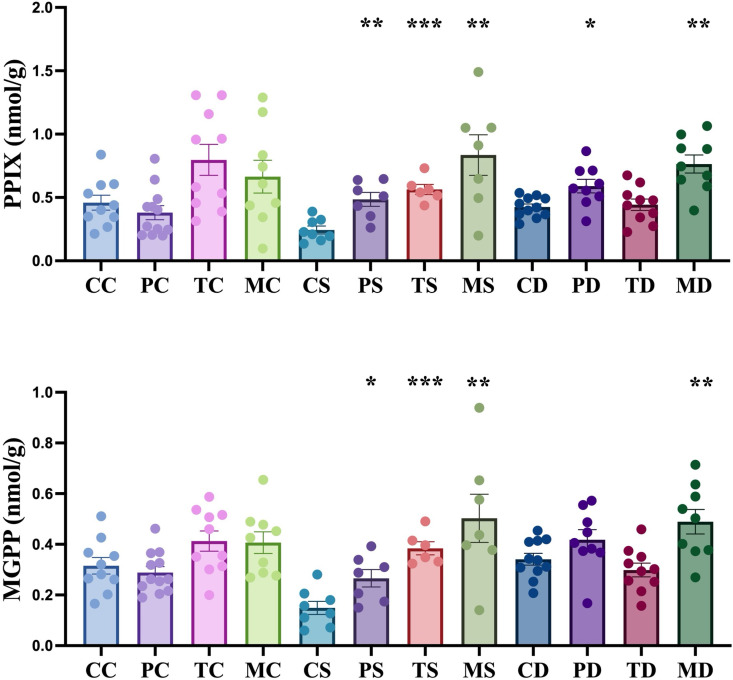
Impact of endophytic fungi on the concentration of protoporphyrin (PPIX) and magnesium protoporphyrin (MGPP) in hemp leaves under non-stress, salinity and drought stress conditions. The first letter; C = control, no fungus, P = with *Pochonia chlamydosporia*, T = with *Trichoderma harzianum*, M = with *Metarhizium robertsii*. The second letter; C = control, non-stress, S = salt stress, D = drought stress. Data were analyzed according to the description of statistical analysis. Statistical differences are shown; *P < 0.05, **P < 0.01, ***P < 0.001. Asterisk indicates significant differences in the tested group when compared to that of the un-inoculated control under the same conditions. The dots represent results of the individual biological replicates. Error bars represent standard error of the means.

As intermediates of the chlorophyll metabolic pathway, porphyrins play an important role in plant resistance to abiotic stress. Under salinity stress, the amounts of protoporphyrin IX (PPIX) and magnesium protoporphyrin (MGPP) in uninoculated control hemp leaves decreased by 47.0% (P < 0.01, unpaired t-test) and 52.7% (P < 0.01, unpaired t-test) compared to control conditions (Fig. 4). However, application of *P. chlamydosporia*, *T. harzianum*, and *M. robertsii* increased the amount of PPIX in hemp by 99.4% (P < 0.01, unpaired t-test), 131.3% (P < 0.0001, unpaired t-test) and 242.9% (P < 0.01, unpaired t-test with Welch’s correction), respectively, compared to the uninoculated control under salinity stress (Fig 4). The amount of MGPP increased by 78.2% (P < 0.05, unpaired t-test), 157.6% (P < 0.0001, unpaired t-test) and 237.0% (P < 0.01, unpaired t-test with Welch’s correction) in hemp with *P. chlamydosporia*, *T. harzianum*, and *M. robertsii* in comparison to the uninoculated control under salinity stress (Fig 4). During drought stress, an increase in PPIX was observed in hemp treated with *P. chlamydosporia* and *M. robertsii* with an increase of 38.8% (P < 0.05, unpaired t-test with Welch’s correction) and 79.8% (P < 0.01, unpaired t-test with Welch’s correction), respectively, in comparison to the uninoculated control with the same conditions. An increase of 43.4% in MGPP (P < 0.01, unpaired t-test) was found in hemp treated with *Metarhizium* compared to uninoculated control under drought stress.

### Transcript levels of stress-responsive transcription factor in hemp leaf

*CsNAC3*, and a reference gene, *EF1α*, were utilized for the quantification of gene expression induced by salt stress in hemp during fungal application of *P. chlamydosporia*, *T. harzianum*, and *M. robertsii.* There was no amplification with fungal genomic DNA and only amplification in hemp genomic DNA through the PCR reaction, which excludes the unspecific amplification of fungal genes in the analysis of plant gene expression.

**Fig 5:**
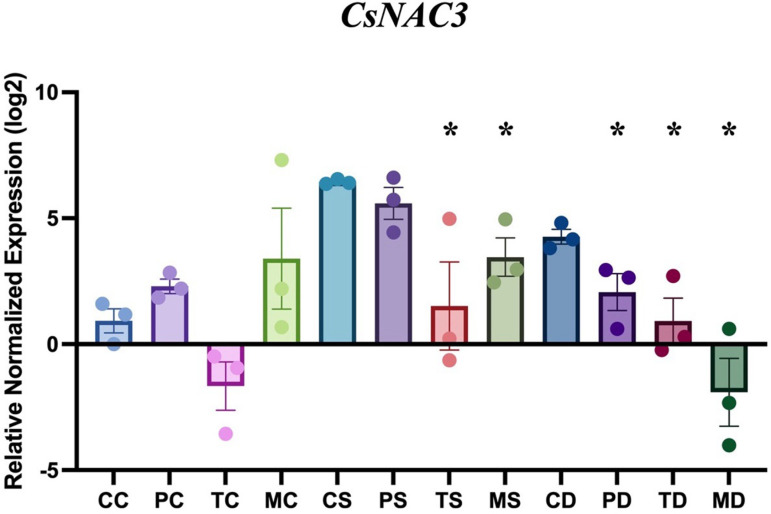
Impact of endophytic fungi on the expression patterns *CsNAC3* in hemp under non-stress, salinity and drought stress conditions. The first letter; C = control, no fungus, P = with *Pochonia chlamydosporia*, T = with *Trichoderma harzianum*, M = with *Metarhizium robertsii*. The second letter; C = control, non-stress, S = salt stress, D = drought stress. Error bars represent standard error of the means. Data were analyzed with standard t-test in Bio-Rad CFX Manager software. Statistical differences are shown; *P < 0.05. Asterisk indicates significant differences in the tested group when compared to that of the un-inoculated control under the same conditions. The dots represent results of the individual biological replicates. Error bars represent standard error of the means.

There were no differences in the relative normalized expression levels of *CsNAC3* in hemp applied with *P. chlamydosporia*, *T. harzianum*, and *M. robertsii* compared to the uninoculated control. These results showed that the expression of *CsNAC3* was not upregulated during interactions between hemp and fungal endophytes under non-stress conditions (Fig 5). Under salinity stress, the relative normalized expression of *CsNAC3* was 5.95-fold (P < 0.001, unpaired t-test) higher in uninoculated hemp leaves compared to the control without stress. When hemp was treated with *T. harzianum* and *M. robertsii* under salinity stress, the relative normalized expression of *CsNAC3* decreased by 76.5% (P < 0.05, unpaired t-test) and 46.4% (P < 0.05, unpaired t-test), respectively, when compared to the uninoculated control under the same treatment. Under drought stress, there was also an increase of relative normalized expression of *CsNAC3* in the uninoculated control with 3.1-fold (P < 0.01, unpaired t-test), higher expression than in non-stress conditions. The application of *P. chlamydosporia*, *T. harzianum*, and *M. robertsii* on hemp resulted in a decrease in the relative normalized expression of *CsNAC3* by 51.6% (P < 0.05), 78.4% (P < 0.05, unpaired t-test) and 144.7% (P < 0.05, unpaired t-test), respectively, when compared to the uninoculated control under drought stress.

## Discussion

Abiotic stresses, such as salinity and drought, is detrimental to crop growth and productivity, and can threaten agricultural sustainability. Plant responses to abiotic stress are well-known [29] and salinity stress can reduce the ability of the plant to take up water, which impedes plant growth. These metabolic changes are similar to those caused by drought [30]. The abiotic stressors, salinity and drought, affect morphological, biochemical, physiological, and molecular processes of plants, including seeds germination, plant growth, and nutrient uptake [31]. Hemp has the characteristics of wide distribution, deep root system, fast growth, high biomass, ease of cultivation and resistance to stress [32]. It can adapt to stress environments such as saline alkali [33]. However, there are relatively large differences in salt tolerance among hemp varieties [34]. Hemp can also survive at exceptionally low levels of water availability in greenhouse conditions [35]. Bacteria and mycorrhizal fungi can alleviate salt or drought stress in hemp plants [36,37]. However, little is known about the association of fungal endophytes with hemp and the alleviation of salinity and drought stresses. In this study, we investigated the influence of fungal endophytes *P. chlamydosporia*, *T. harzianum*, and *M. robertsii* on hemp under salinity and drought stress. Our results suggested that the application of these fungal endophytes ameliorated the negative effects of hemp growth under a high-salinity environment and *M. robertsii* also alleviated stress-induced growth inhibition drought stress.

In this study, the fungal endophytes, *M. robertsii, T. harzianum* and *P. chlamydosporia*, mitigated the reduction in hemp growth under salinity stress as measured by leaf, stem, and root weight (Fig 1 and Fig 2). Similar effects have been observed on other host plants and fungal endophytes such as soybean with *Metarhizium anisopliae* LHL07 [38], tomato with *Metarhizium pinghaense* AAUBC-M26 [39], and rice with *M. anisopliae* MetA1 [40]. As traditional biocontrol agents, the mitigating effects of *T. harzianum* application on plant growth under salinity stress was observed in tomato [41], chickpea [42], Indian mustard [43] and sweet sorghum [44]. In this study, we also observed an increase in leaf pigments in *T. harzianum* colonized hemp under salinity stress (Fig S1). *P. chlamydosporia* was reported to improve plant growth under normal conditions in barley [12], tomato and lettuce [45], *Arabidopsis* [46], banana [47], and hemp [18]. Here we also report the effects of *P. chlamydosporia* under salinity stress tolerance in hemp. We observed an improvement of above-ground growth (leaf weight and stem weight) in hemp plants with *M. robertsii* during drought stress. A significant positive relationship between the intensity of *M. robertsii* root colonization and maize height under a deficit water treatment was observed [48]. We did not observe an improvement of hemp growth with the application of *P. chlamydosporia* or *T. harzianum* under drought stress in this study. Although it was reported that *T. harzianum* Th-56 improved drought tolerance in rice [49].

Oxidative damage to proteins, DNA, and lipids occurs in plants during stress responses, and can affect plant immune responses and changes in stomatal behavior, and can initiate programmed cell death [2]. As one of the final products of unsaturated fatty acid peroxidation in phospholipids, malondialdehyde (MDA) is an indicator of lipid peroxidation in plants under salt stress [50]. A higher MDA content can result in damage to the plant cell membrane. The increased oxidative stress represented by increased MDA levels together with enzyme activity of anti-oxidative enzymes were observed in ‘Bama’ hemp under the salinity stress of 140 mM NaCl [51] and 40 mM of CaCl_2_ [52]. Accumulation of MDA in salt-treated uninoculated hemp implies that plants were suffering from stress (Fig 3). The levels of MDA decreased in hemp colonized by *M. robertsii*, *T. harzianum* and *P. chlamydosporia* compared with the uninoculated control under salinity stress, suggesting that the presence of these fungal endophytes is correlated with a reduction in a stress response. A similar reduction in MDA under salinity stress was also reported in soybean colonized with *M. anisopliae* LHL07 [38], chickpea with *T. harzianum* Th-14 [42] and Indian mustard with *T. harzianum* [43]. No differences in the amount of MDA were detected during fungal endophyte application on hemp compared to the uninoculated control under non-stress conditions, which suggests there was no lipid peroxidation stress caused by the application of these fungi. Similar results were reported in soybean with *M. anisopliae* LHL07 [38], chickpea with *T. harzianum* Th-14 [42], Indian mustard with *T. harzianum* [43], and the endophyte *Epichloë festucae* var. *lolii* colonized perennial ryegrass [53], *Epichloë coenophiala* colonized tall fescue [54], and *Neotyphodium gansuense* colonized drunken horse grass [55].

Porphyrins play important roles during the processes of light harvesting, detoxification and signal transduction [56]. PPIX is a common precursor in the chlorophyll and heme biochemical pathways in plants and MGPP is involved in the insertion of Mg^2+^ into PPIX. MGPP was reported as a signalling molecule in one of the signalling pathways between the chloroplast and nucleus [57]. The roles of porphyrins during plant abiotic stress are not well elucidated. They are assumed to be involved in tetrapyrrole-dependent plastid-to-nucleus signaling pathways [58], or related to oxidative stress homeostasis in the plant [59]. A reduction in PPIX and MGPP was observed in rice under salinity stress and drought stress [60,61]. Overexpression of porphyrin protected transgenic plants from drought-induced cytotoxicity and demonstrated that porphyrin metabolism and signaling during water-related stress are important for dehydration protection of the plant cell [61]. In this study, reductions in PPIX and MGPP were observed in hemp during salinity stress (Fig 4). The application of *M. robertsii*, *T. harzianum* and *P. chlamydosporia* recovered PPIX and MGPP concentrations during salt stress, associated with the regulation of oxidative stress [59] or tetrapyrrole-dependent plastid-to-nucleus signaling pathways [58] in hemp. There were no differences in the amounts of PPIX and MGPP in uninoculated hemp under normal conditions and drought stress, which may result from the relatively high drought tolerance of hemp [35]. However, recovery of PPIX and MGPP was observed in hemp with *M. robertsii* under drought stress, which was correlated to an improvement of above-ground growth in hemp.

*CsNAC3*, a hemp transcription factor, was up-regulated in hemp under salinity and drought stress compared to control conditions [62,63], which was similar to what we observed in this study (Fig 5). The overexpression of transcription factors induced by salt stress in hemp enhances salt tolerance in tobacco [16]. However, in this study, we observed a reduction in expression of *CsNAC3* in hemp colonized by *M. robertsii* and *T. harzianum* under salt stress, and *M. robertsii*, *T. harzianum* and *P. chlamydosporia* under drought stress, compared to the controls. Considering that different signals can regulate the expression of transcription factors, such as MAPK cascades, phytohormones, calcium ion and reactive oxygen species [64], there might be other stress sensor factors influenced by fungal endophytes in hemp under abiotic stress. The downregulation of *CsNAC*3 during fungal application under abiotic stress may result from reduced oxidative stress in hemp, rather than the corresponding factor in the plant influenced by fungal endophytes. The regulation of this, and other transcription factors, in the presence of fungal endophytes and abiotic stress is a potentially rich topic of research.

Under non-stress conditions, an increase in the weight of hemp leaf, stem and root was observed during application of *P. chlamydosporia* (Fig 2). Similar results were observed in hemp growth during application of this fungus [18]. However, growth improvement by *M. robertsii* was only observed in hemp leaf weight, not stem weight and root weight. *T. harzianum* application in hemp showed no influence on hemp growth, which differs from previous research of *T. harzianum* T-22 with cannabis varieties ‘Fedora 17’ and ‘Felina’ [65]. These differing results could potentially be explained by responses of different hemp varieties (i.e., fibre type variety in this study, and oilseed type variety in a previous study).

The research in this study provides some promising results in the application of fungal endophytes as a prophylactic against abiotic perturbations during the growth of hemp in greenhouses. The elucidation of mechanisms in the alleviation of the abiotic stress in plants during endophytic colonization will increase our understanding of this symbiotic association and the application of fungal endophytes in agricultural plants for resilience against long-term climatic changes and extending the cultivatable area to saline environments. Fiber and seed type of hemp were reported to respond differently to salt-alkali stress in seedling growth and physiological indices [20]. Seed germination of hemp cultivars also responded differently to the stress of salt type and concentration [66]. The ANKA strain used in this study is utilized in the production of hemp fibre. Therefore, future research could assess the amelioration of plant growth under different abiotic stresses by fungal endophytes in different varieties of hemp and under field conditions. With the development of tools and applications of proteomics in plants [67], the future research of proteomic and metabolomic analysis [68–72] of hemp responses to abiotic stresses and the role of fungal endophytes in these responses could provide more details and deepen our understanding of the beneficial relations between fungal endophytes and plants.

## Conclusions

Here we showed that endophytic association of *Pochonia*, *Trichoderma* and *Metarhizium* can mitigate the detrimental effects of NaCl stress in hemp. The alleviation of stresses by fungal endophytes was associated with a reduction in lipid oxidative stress, production of porphyrins and expression of a stress related transcription factor. This research provides the potential utility of environmentally friendly biofertilizers that can boost the growth of economically significant plants under saline environmental conditions on marginal agricultural lands.

## Supporting information

S1 Fig Impact of endophytic fungi on hemp leaf pigments, under non-stress, salinity and drought stress conditions. The first letter; C = control, no fungus, P = with *Pochonia chlamydosporia*, T = with *Trichoderma harzianum*, M = with *Metarhizium robertsii*. The second letter; C = control, non-stress, S = salt stress, D = drought stress. Data were analyzed according to the description in statistical analysis. Statistical differences are shown; *P < 0.05, **P < 0.01. Asterisk indicates significant differences in the tested group when compared to that of the un-inoculated control under the same conditions. The dots represent results of the individual biological replicates. Error bars represent standard error of the means.
(TIFF)

S2 Fig Impact of endophytic fungi on hemp leaf water content, under non-stress, salinity and drought stress conditions. The first letter; C = control, no fungus, P = with *Pochonia chlamydosporia*, T = with *Trichoderma harzianum*, M = with *Metarhizium robertsii*. The second letter; C = control, non-stress, S = salt stress, D = drought stress. Data were analyzed according to the description in statistical analysis Statistical differences are shown; ***P < 0.01. Asterisk indicates significant differences in the tested group when compared to that of the un-inoculated control under the same conditions. The dots represent results of the individual biological replicates. Error bars represent standard error of the means.
(TIFF)

S3 Fig Impact of endophytic fungi on the soil electrical conductivity of hemp plant, under non-stress, salinity and drought stress conditions. The first letter; C = control, no fungus, P = with *Pochonia chlamydosporia*, T = with *Trichoderma harzianum*, M = with *Metarhizium robertsii*. The second letter; C = control, non-stress, S = salt stress, D = drought stress. Data were analyzed according to the description in statistical analysis. No Statistical differences are shown. The dots represent results of the individual biological replicates. Error bars represent standard error of the means.
(TIFF)

S4 Fig Impact of endophytic fungi on soil humidity, under non-stress, salinity and drought stress conditions. The first letter; C = control, no fungus, P = with *Pochonia chlamydosporia*, T = with *Trichoderma harzianum*, M = with *Metarhizium robertsii*. The second letter; C = control, non-stress, S = salt stress, D = drought stress. Data were analyzed according to the description in statistical analysis. Statistical differences are shown; *P < 0.05, **P < 0.01, ***P < 0.001. Asterisk indicates significant differences in the tested group when compared to that of the un-inoculated control under the same conditions. The dots represent results of the individual biological replicates. Error bars represent standard error of the means.
(TIFF)
