# Supplementary figures and images for: The endophytic fungi Metarhizium, Pochonia, and Trichoderma, improve salt tolerance in hemp (Cannabis sativa L.)
Source: PLoS One. 2025 Jun 11;20(6):e0325559. doi: 10.1371/journal.pone.0325559 (PMC12157123; doi:10.1371/journal.pone.0325559)

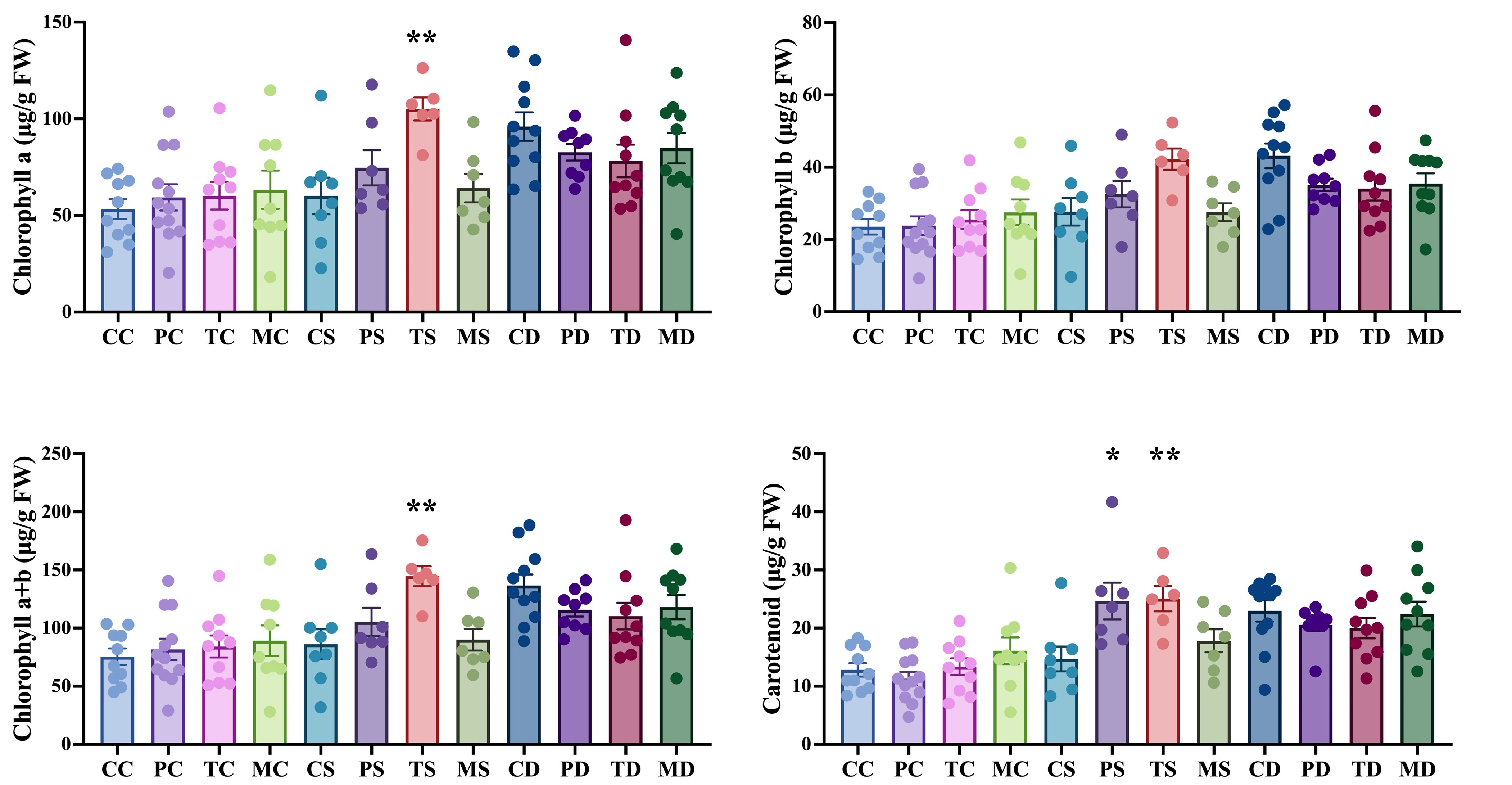

Supplement: S1 Fig — The first letter; C = control, no fungus, P = with Pochonia chlamydosporia, T = with Trichoderma harzianum, M = with Metarhizium robertsii. The second letter; C = control, non-stress, S = salt stress, D = drought stress. Data were analyzed according to the description in statistical analysis. Statistical differences are shown; *P < 0.05, **P < 0.01. Asterisk indicates significant differences in the tested group when compared to that of the un-inoculated control under the same conditions. The dots represent results of the individual biological replicates. Error bars represent standard error of the means. (TIFF) [file pone.0325559.s001.tiff]

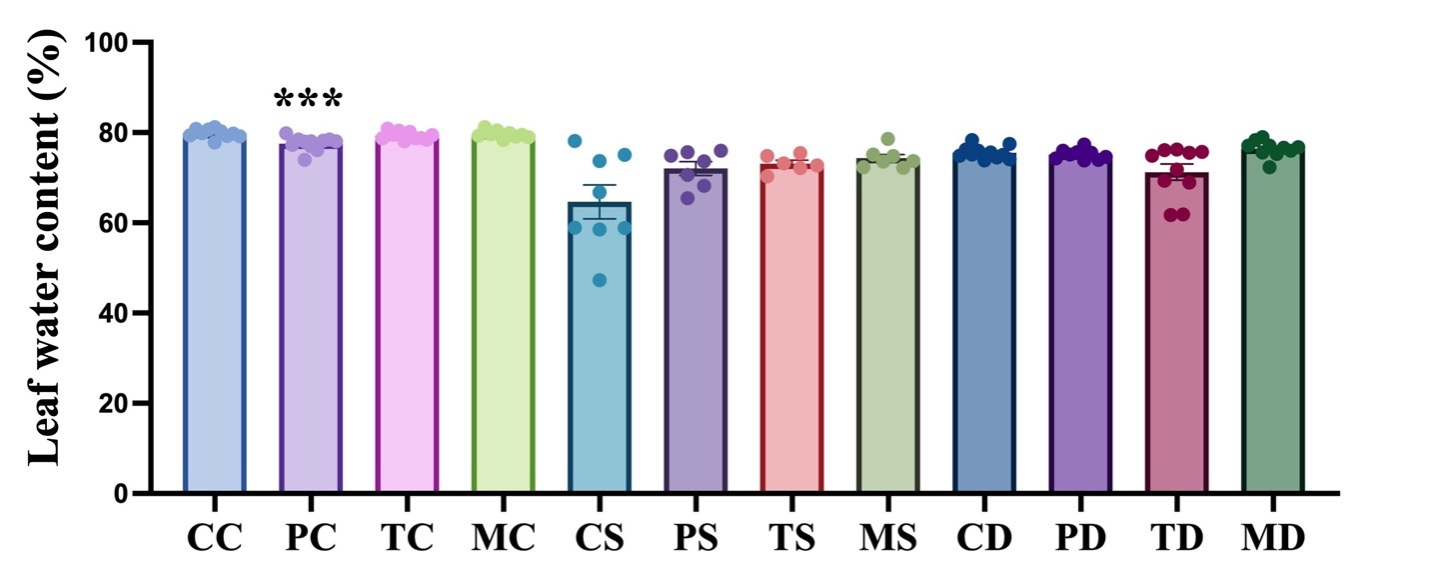

Supplement: S2 Fig — The first letter; C = control, no fungus, P = with Pochonia chlamydosporia, T = with Trichoderma harzianum, M = with Metarhizium robertsii. The second letter; C = control, non-stress, S = salt stress, D = drought stress. Data were analyzed according to the description in statistical analysis Statistical differences are shown; ***P < 0.01. Asterisk indicates significant differences in the tested group when compared to that of the un-inoculated control under the same conditions. The dots represent results of the individual biological replicates. Error bars represent standard error of the means. (TIFF) [file pone.0325559.s002.tiff]

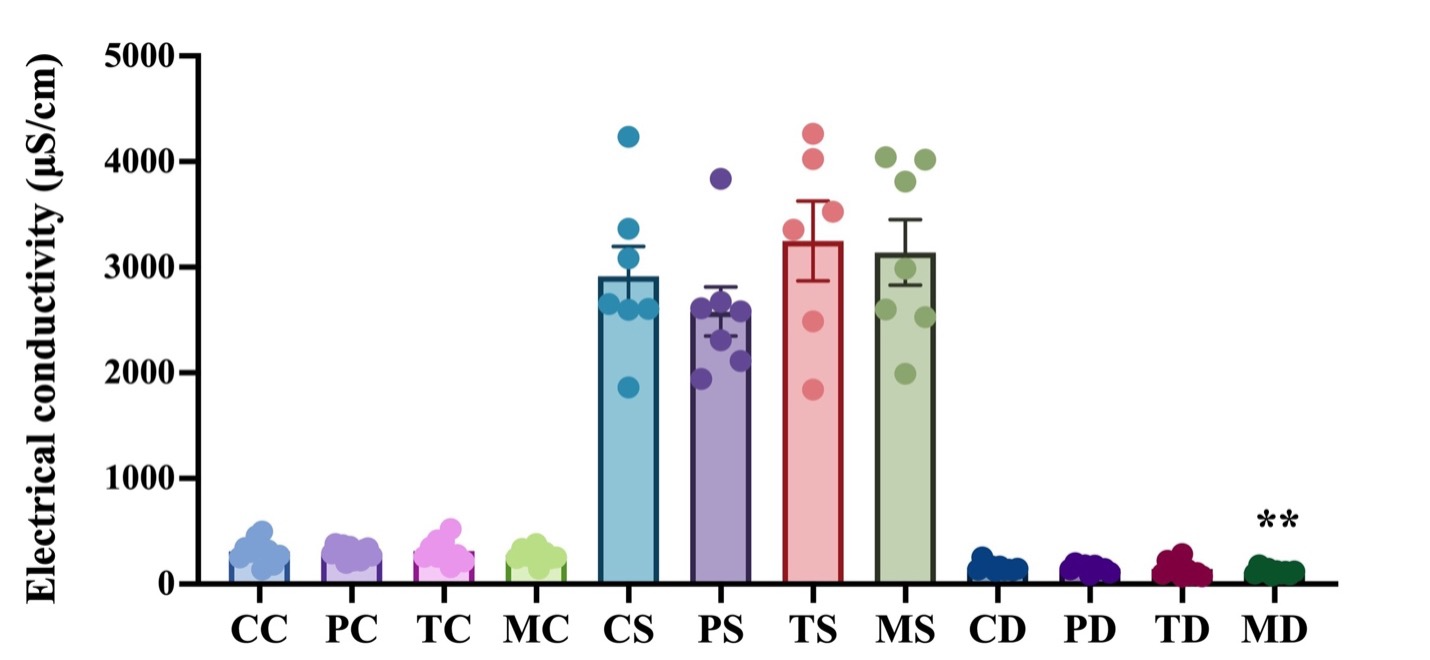

Supplement: S3 Fig — The first letter; C = control, no fungus, P = with Pochonia chlamydosporia, T = with Trichoderma harzianum, M = with Metarhizium robertsii. The second letter; C = control, non-stress, S = salt stress, D = drought stress. Data were analyzed according to the description in statistical analysis. No Statistical differences are shown. The dots represent results of the individual biological replicates. Error bars represent standard error of the means. (TIFF) [file pone.0325559.s003.tiff]

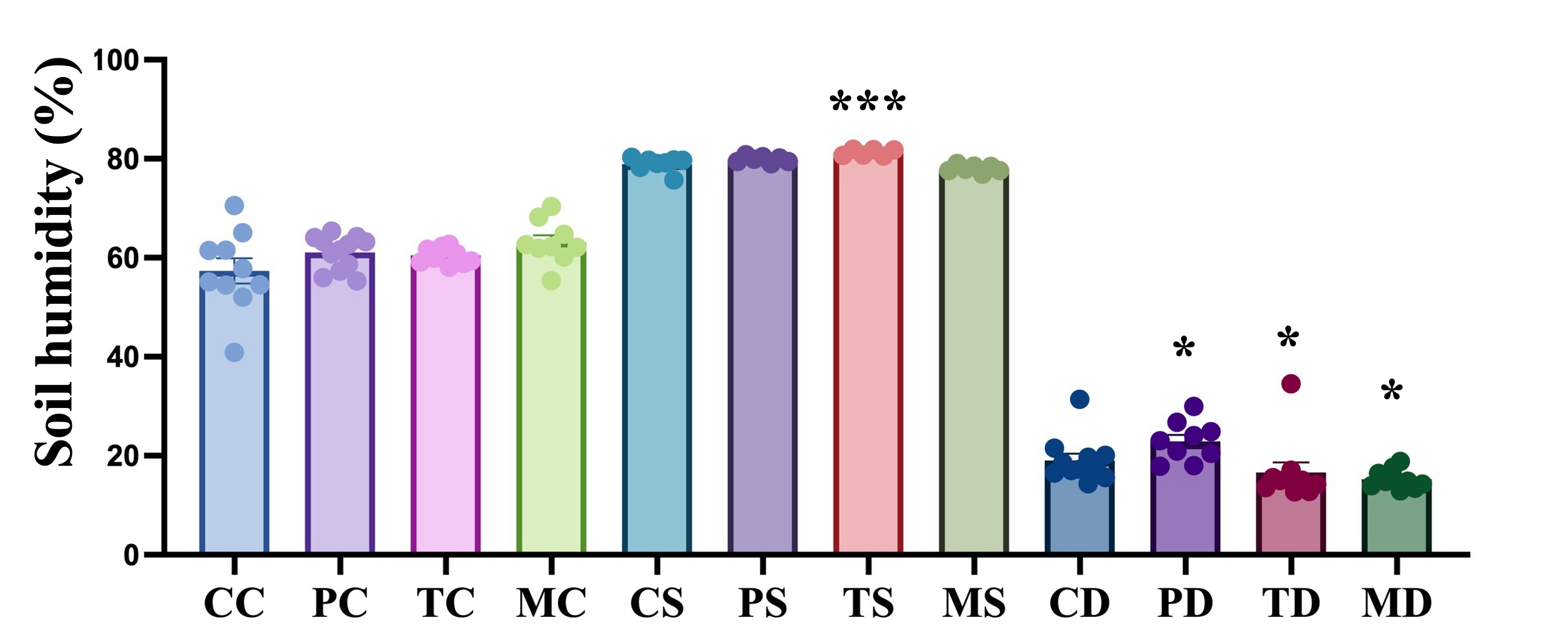

Supplement: S4 Fig — The first letter; C = control, no fungus, P = with Pochonia chlamydosporia, T = with Trichoderma harzianum, M = with Metarhizium robertsii. The second letter; C = control, non-stress, S = salt stress, D = drought stress. Data were analyzed according to the description in statistical analysis. Statistical differences are shown; *P < 0.05, **P < 0.01, ***P < 0.001. Asterisk indicates significant differences in the tested group when compared to that of the un-inoculated control under the same conditions. The dots represent results of the individual biological replicates. Error bars represent standard error of the means. (TIFF) [file pone.0325559.s004.tiff]
